# Notes on the Examination of the Sputum, Vomit, Fæces, and Urine

**Published:** 1891-06

**Authors:** 


*Notes on the Examination of the Sputum, Vomit, Fæces, and Urine.* By SIDNEY COUPLAND, M.D., F.R.C.P. London: H. K. LEWIS. 1891.

The author states that these memoranda were originally compiled for the use of the students of his class. They comprise directions confined to the most important points in the examination of the above excretions; and these have been made with judgment, and are clear and accurate. Such notes are serviceable to students, if they bear in mind that they are to be used only as a means, by frequent reference in their daily work, of more readily acquiring the elementary facts in the clinical examination of the excretions, and not as a substitute for thoroughly committing these facts to memory.

